# Synergistic effect in the co-extraction of *Ginseng* and *Schisandra* protein

**DOI:** 10.3389/fnut.2024.1482125

**Published:** 2024-10-31

**Authors:** Han Zhang, Haidong Wang, Hongyan Zhou, Jialin Shi, Zhiqiang Wan, Guangzhe Li, Mingming Yan

**Affiliations:** ^1^Changchun University of Chinese Medicine, Changchun, China; ^2^Jinlin Provincial Science and Technology Innovation Center of Health Food of Chinese Medicine, Changchun, China

**Keywords:** synergistic effect, co-extracted protein, physicochemical properties, functional properties, antioxidant properties

## Abstract

**Introduction:**

*Ginseng* and *Schisandra* are traditional Chinese plants that have been used in culinary practices and are renowned for their immune-boosting properties. In Chinese medicine, *Ginseng* and *Schisandra* are frequently used together as a clinical pair to mutually enhance their effect, producing a synergistic effect when consumed in combination. However, the underlying mechanism of their synergistic effect remains uncertain. Therefore, this study investigates the synergistic effect of *Ginseng*-*Schisandra* in terms of macromolecular proteins.

**Methods:**

We used a dual-protein research methodology combined with co-extraction techniques to obtain the co-extracted protein of ginseng and Schisandra. We then compared the physicochemical and functional properties and antioxidant activities of co-extracted protein (COP), simple mixed protein (SMP), *Ginseng* protein (PGP), and *Schisandra* protein (SCP).

**Results:**

Generally, PGP and SCP are considered as functional food with antioxidant activity. COP are composite proteins with a shared internal structure that are combined by *Ginseng* and *Schisandra* proteins, while SMP are simple mixtures of PGP and SCP. Free radical scavenging experiments indicated that COP exhibited the highest scavenging ability for hydroxyl radicals (98.89%), 1,1-diphenyl-2-picrylhydrazyl (DPPH) radicals (85.95%), and 2,2′-azinobis-(3-ethylbenzthiazoline-6-sulfonate) (ABTS+) radicals (42.69%). *In vitro*, COP significantly reduced the accumulation of reactive oxygen species (ROS) and malondialdehyde (MDA), while increasing intracellular levels of superoxide dismutase (SOD), glutathione peroxidase (GSH-Px), catalase (CAT), and lactate dehydrogenase (LDH) levels in HepG2 cells.

**Discussion:**

The comparative results of the macromolecular proteins reveal that COP contributes to the synergistic effect of Ginseng-Schisandra and indicate the advantages of co-extraction in protein production, suggesting the potential application of COP in the food industry.

## 1 Introduction

Protein is an important nutrient for humans, playing a key role in human growth, development, and disease resistance. With the rapid development of food engineering, there has been an increasing focus on developing new protein rich foods rich that are not only nutrient-dense but also multifunctional.

In recent years, dual-protein systems have gained attention due to their enhanced nutritional and functional properties. These systems can compensate for some of the deficiencies found in single-protein sources, making them highly promising in the field of nutritional food supplements. As a result, dual proteins have emerged as a research hotspot in the development of new protein-based foods.

*Ginseng* and *Schisandra* are rich in nutrients and are widely used as tonic foods in Chinese traditional customs. Both plants are well-known for their dual roles as medicinal and edible substances in China. They are commonly used in medicinal diets. *Ginseng* (1) and *Schisandra* (2), are often combined as a “drug pair” in such diets to achieve synergistic effects, enhancing efficacy, reducing fatigue, and improving immunity. In view of the rich nutritional components and wide edible value of Ginseng and Schisandra, numerous scholars have conducted in-depth research on *Ginseng*-*Schisandra* (3, 4). For example, Studies have detected lignans were detected in Ginseng-Schisandra granules by High Performance Liquid Chromatography (HPLC) (5). The small molecule lignans and saponins of the Ginseng-Schisandra drug pair have been reported to have therapeutic effects on rats with Alzheimer’s disease (AD) (6). The lignans in *Ginseng*-*Schisandra* can pass through the blood–brain barrier and play a role in protecting the central nervous system (7). The aforementioned studies have shown that the lignans and phenolic acids in *Ginseng*-*Schisandra* have anti-oxidation, anti-inflammation, and liver protection effects, as well as obvious synergistic enhancement effects. However, these lignans and phenolic acids are small molecular compounds, most of which are not easily soluble in water, and the extraction yield is low. The safety of these compounds also needs to be further verified. Moreover, ensuring their bioavailability is difficult, and there are certain limitations to their applications. Our previous studies have shown that the *Ginseng* and *Schisandra* proteins are rich in amino acids and are considered to be nutritious high-quality protein foods (8, 9). Given the synergistic effect of *Ginseng* and *Schisandra* as a “drug pair” in medicinal diets, determining how to effectively extract and separate the *Ginseng*-*Schisandra* double protein by co-extraction technology and comparing it with the single proteins of the two in terms of physical and chemical properties, functional characteristics and antioxidant activity is of key research significance.

Co-extraction is a common mixed double protein extraction method, whereby, the double protein is prepared by the isoelectric precipitation of the two mixture materials. The double protein prepared by this method is also denoted as the co-extracted protein (10). In the extraction process, by adjusting the pH, the proteins from the two materials are reassembled by unfolding, interacting, and refolding to obtain the co-assembled proteins from both sides (11, 12). In recent years, co-extraction technology has been gradually applied in double protein extraction. Scholars have reported that the co-extracted proteins isolated from Tilapia-Soybean (13) and Pea-Grass Carp (14) by co-extraction technology have rich nutritional qualities, and their physical, chemical, and functional properties and biological activities are enhanced compared to the single materials. Thus, co-extraction technology can effectively and feasibly mix proteins from different sources and overcome the lack of essential amino acids, insufficient nutritional value, and poor functional characteristics of single proteins (15).

This study used co-extraction technology to prepare the *Ginseng*-*Schisandra* co-extracted protein. In particular, the *Ginseng*-*Schisandra* co-extracted protein was taken as the research object, with *Ginseng* protein (PGP) and *Schisandra* protein (SCP) as references. Through a comparative study of the physical, chemical, and functional properties and biological activity of the *Ginseng*-*Schisandra* simple mixed protein (SMP) and co-extracted protein (COP), the synergistic effect of COP was discussed. The study provides a scientific basis and theoretical reference for the exploration of co-extracted protein foods with comprehensive nutrients, ideal functional characteristics, and superior biological activity.

## 2 Materials and methods

### 2.1 Materials and reagents

The medicinal herbs *Ginseng* and *Schisandra* were purchased from the Affiliated Hospital of Changchun University of Traditional Chinese Medicine, (Jilin, China). Standard protein markers, o-phenanthroline (o-diazepine), 1,1-diphenyl-2-picrylhydrazyl (DPPH), 2,2′-azino-bis-(3-ethylbenzothiazoline-6-sulfonic acid) (ABTS), and Vitamin C (ascorbic acid) were of analytically pure grade and obtained from Shanghai Preferred Bio-technology Co., Ltd. (Shanghai, China). The live/dead cell staining kit was obtained from Beyotime Biotechnology Co., Ltd. (Shanghai, China). The cellular Reactive Oxygen Species (ROS) detection kit was obtained from Beyotime Biotechnology Co., Ltd. (Shanghai, China). The Annexin V-FITC/PI dual staining apoptosis detection kit was obtained from Beyotime Biotechnology Co., Ltd. (Shanghai, China). Human hepatocellular carcinoma cells (HepG2) were obtained from Wuhan Pricella Biotechnology Co., Ltd. (Wuhan, China). Fetal bovine serum and trypsin were obtained from Gibco Ltd. (United States). CCK-8 and PBS were obtained from Beijing Bioss Co., Ltd. (Beijing, China). Catalase (CAT), glutathione peroxidase (GSH-Px), superoxide dismutase (SOD), malondialdehyde (MDA) and lactate dehydrogenase (LDH) test kits were obtained from Sino Best Biological Technology Co., Ltd. (Shanghai, China).

### 2.2 Sample preparation

The *Ginseng* and *Schisandra* were crushed and weighed according to the mass ratio of 6:1. The weighed *Ginseng* and *Schisandra* powder were placed into a beaker and mixed with distilled water at a ratio of 1:20 (w/v). The pH value was adjusted to 9.0 with 1 mol/L NaOH solution. The solution was stirred in a water bath at 35°C for 3 h and subsequently centrifuged (8,000 r/min, 10 min). The precipitate was discarded and the pH value of the supernatant was adjusted to 3.5 with 1 mol/L HCL solution. The solution was allowed to stand at 4°C for 2 h and subsequently centrifuged (8,000 r/min, 10 min). The supernatant was discarded and the precipitate was dissolved in a small amount of distilled water. The pH value was adjusted to a neutral level with 1 mol/L NaOH solution and placed into a dialysis bag (molecular weight of 8,000–14,000). The bag was dialyzed at 4°C for 48 h and the distilled water was replaced every 2 h. The liquid in the dialysis bag was freeze-dried. The freeze-dried material was denoted as COP. The *Ginseng* and *Schisandra* powder weighed and PGP and SCP were obtained according to the above process. The yields of PGP and SCP were then calculated and used to determine the ratio of PGP to SCP at the Ginseng-Schisandra powder mass ratio of 6:1. Appropriate amounts of PGP and SCP were weighed in proportion and mixed to obtain SMP.

### 2.3 Physicochemical properties

#### 2.3.1 Sodium dodecyl sulfate–polyacrylamide gel electrophoresis

Protein samples were prepared at a concentration of 5 mg/mL and centrifuged at 8,000 r/min for 10 min. The supernatant was mixed with reduced and non-reduced protein sampling buffer at a 1:1 ratio. The mixture was then cooled in a boiling water bath for 5 min and electrophoresed by sodium dodecyl sulfate–polyacrylamide gel electrophoresis (SDS–PAGE) using polyacrylamide concentrates and separator gels with concentrations of 5 and 12%, respectively. The gel was stained with Caulmers Brilliant Blue R-250 at room temperature for 1 h and decolorized multiple times with decolorizing solution until the background became transparent and the bands were clear. Finally, the gel was scanned and imaged using a gel imager (iBrightFL1000, Thermo Fisher Scientific, United States).

#### 2.3.2 Ultraviolet spectroscopy

Ultraviolet (UV) spectra were obtained using a UV-2550 ultraviolet–visible (UV–Vis) spectrophotometer (Shimadzu, Tokyo, Japan) according to the method described by Zhao et al. (16). The samples (2 mg/mL) were prepared with distilled water. The distilled water was used as the reference. The UV absorption spectra were recorded at the wavelength range of 200–500 nm with a slit width of 5 nm.

#### 2.3.3 Fourier transform infrared spectroscopy

The secondary structures of the four proteins were analyzed using Fourier transform infrared (FT-IR) spectroscopy following the method of Xie et al. (17). A total of 2 mg of the sample was accurately weighed and mixed with 200 mg of KBr. The resulting mixture was milled and pressed into tablets, which were placed on a sample holder for spectral scanning. The scanning range was 4,000–400 cm^−1^, with a spectral resolution of 4 cm^−1^ and 32 scans per measurement.

#### 2.3.4 Circular dichroism spectroscopy

The structure of the four proteins was analyzed using circular dichroism (CD) spectroscopy according to the method described by Yuan et al. (18). A total of 1 mg of protein lyophilized powder was weighed and dissolved in distilled water to obtain a 100 mL solution. The solution was then placed in a 0.1 cm quartz cuvette and scanned using a CD spectrometer (J-815, JASCO Corporation, Japan) with the following parameters: scanning wavelength range of 185–260 nm, scanning speed of 100 nm per min, bandwidth of 1.0 nm, response time of 0.50 s, and step resolution of 1.0 nm.

#### 2.3.5 Differential scanning calorimetry

Differential scanning calorimetry (DSC) was employed to analyze the thermal behavior of the samples with DSC 250 (TA Instruments, United States), following the method of Sadaf with minor adjustments (19). In brief, 2 mg of powder was placed in an aluminum disk and sealed with an aluminum cover. The initial scanning temperature was set at 25°C and increased to 180°C at a heating rate of 5°C/min.

#### 2.3.6 Scanning electron microscopy

The lyophilized samples were fixed on a sample stage, sprayed with gold, and coated with a thin film of gold in a vacuum evaporator. Scanning electronic microscopy (SEM) (S–3400N microscope, Hitachi, Tokyo, Japan) was then used to observe the microstructure of the samples and obtain images at an accelerating voltage of 5 kV and a 200× magnification.

#### 2.3.7 Protein–protein interaction

By measuring the dissolution of protein in six different extracts, the effect of the protein type on protein solubility was analyzed following the method of Tian et al. (20). Six solvents were prepared by dissolving the four proteins. These solvents were denoted as B1 (0.03 M PBS), B2 (0.03 M PBS +1% (w/v) SDS), B3 (0.03 M PBS + 6 M Urea), B4 (0.03 M PBS +2% (w/v) 2-Mercaptoethanol), B5 (0.03 M PBS +1% (w/v) SDS + 2% (w/v) 2-Mercaptoethanol), and B6 (0.03 M PBS + 1% (w/v) SDS + 6 M Urea). The protein content of the supernatant was determined by centrifuging the proteins in each of the solutions at 10,000 × g for 10 min.

#### 2.3.8 Free sulfhydryl and disulfide bond

The contents of sulfhydryl (SH) groups and disulfide (SS) bonds are important in numerous protein foods. Free SH was determined following the method of Chen et al. (21). In brief, a protein sample (50 mg) was dissolved in 15 mL of Tris-Gly buffer (8 mol/L urea, 50 μL of Ellman’s reagent). The mixture was incubated in the dark at 25°C for 1 h and then centrifuged. The absorbance value of the supernatant was determined at 412 nm.

To determine the total SH content, a protein sample (25 mg) was dissolved in 15 mL of Tris-Gly (8 mol/L urea, 100 μL of *β*-ME). The mixture was incubated at 25°C for 1 h. Following this, 15 mL of 12% trichloroacetic acid was added and the mixture was centrifuged. The sediment was washed three times with 15 mL of 12% trichloroacetic acid and centrifuged again. The sediment was resuspended in 15 mL of Tris-Gly (8 mol/L urea). The absorbance value of the solutions containing 50 μL of Ellman’s reagent was determined at 412 nm. The SH ( μM/g) and SS $\left(\mu M/g\right)$ contents were then calculated as:


$$SH=\frac{\left(73.53\times A_{412}\times D\right)}{C}$$

$$SS=\frac{\mathrm{Total}\;SH\;\mathrm{value}-\mathrm{Free}\;SH\;\mathrm{value}}{2}$$

where 73.53 is a coefficient; *A*_412_ is the absorbance; *D* is the dilution factor; and *C* is the protein concentration (mg/mL).

#### 2.3.9 Amino acid composition analysis

To analyze the amino acid composition in the proteins, 20 mg of each protein sample was weighted and placed in a vacuum hydrolysis tube. Following this, 4 mL of 6 mol/L HCl solution was added to hydrolyze the four protein samples. The tube was then filled with nitrogen and sealed. The reaction was performed at 110°C ± 1°C for 24 h. After the reaction, the hydrolysis of the samples was dried at 50°C and dissolved in the automatic amino acid analyzer (A300, membra Pure GmbH, Germany). Amino acid composition is expressed as 1 g/100 g protein.

### 2.4 Functional characteristics

#### 2.4.1 Solubility

Protein solution was prepared at 0.5% (W/V). The pH was adjusted to 2.0, 4.0, 6.0, 8.0, and 10.0 and stirred for 30 min after centrifuging at 8,000 r/min for 20 min. Bovine serum albumin (BSA) was used as the standard and the protein content in the supernatant was measured using the Lowry method at 750 nm. The solubility (%) was calculated as follows:


$$\mathrm{Solubility}=\frac{C}{C_{0}}\times 100$$

where *C* and *C*_0_ are the protein contents in the sample after and before centrifugation (mg), respectively.

#### 2.4.2 Water holding capacity

To determine the water holding capacity (WHC) the protein samples (1.5 g) were added to 10 mL of distilled water and shaken for 2 min. The mixture was then left to stand for 80 min at 25°C and centrifuged. The supernatant was decanted and the weight was recorded. The WHC (%) value of the sample was calculated as follows:


$$WHC=\frac{m_{2}-m_{1}}{m}\times 100$$

where *m* is the quality of the protein sample (g); *m*_1_ is the quality of the distilled water (g); and *m*_2_ is the quality of the supernatant (g).

#### 2.4.3 Oil adsorption capacity

For the determination of the oil adsorption capacity (OAC), the protein samples (0.5 g) were placed in a centrifuge tube containing 5 g of soybean oil. The mixture was left to stand for 30 min, mixed every 3 min, and centrifuged. The weight of the upper oil was then recorded. The OAC (%) value was calculated as follows:


$$OAC=\frac{m_{2}-m_{1}}{m}\times 100$$

where *m* is the quality of the protein sample (g); *m*_1_ is the quality of the soybean oil (g); and *m*_2_ is the quality of the upper oil (g).

#### 2.4.4 Emulsification

A 0.5% protein solution was prepared, and the pH was adjusted to 2.0, 4.0, 6.0, 8.0, and 10.0. The solutions were stirred and dissolved for 30 min. The protein solutions (6 mL) were mixed with soybean oil (2 mL) and emulsified with the high-speed homogenizer (Shanghai Specimen Model Co., Shanghai, China) for 1 min (12,000 rpm). Subsequently, 20 μL of each emulsion was collected from the bottom container and dispersed in 4 mL of 0.1% SDS. The absorbance (A_0_) was measured at a wavelength of 500 nm. After 10 min, 20 μL of the emulsion was again collected from the bottom container and was dispersed in 4 mL of 0.1% SDS. The absorbance (A_10_) was tested at a wavelength of 500 nm by the method of Abir et al. (22). The blank control was 0.1% SDS. Emulsification efficiency (EAI, $m^{2}/g)$ and emulsion stability (ESI, min) at various pH levels were assessed as follows:


$$EAI=\frac{2\times 2.303\times A_{0}\times D}{C\times \phi \times 10^{4}}\;\times 100\text{,}$$

$$ESI=\frac{A_{0}}{A_{0}-A_{10}}\times \Delta t\text{,}$$

where *D* is the dilution ratio (200), C denotes the protein concentration (5 mg/mL), ϕ represents the volume fraction of oil in the emulsion (0.25), *L* refers to the optical cuvette path length (1 cm), and ∆*t* indicates the measurement interval (10 min).

### 2.5 Antioxidant activities

#### 2.5.1 Free radical scavenging activity

##### 2.5.1.1 Hydroxyl radical scavenging activity

The hydroxyl radical scavenging activity was evaluated following the method of Ren et al. (23), with some modifications. In brief, 1 mL of the samples at different concentrations (0.5 mg/mL, 1.0 mg/mL, 1.5 mg/mL, 2.0 mg/mL, 2.5 mg/mL), 1 mL FeSO_4_ (1.8 mmol/L), and 1 mL of 1.8 mmol/L of H_2_O_2_ were allowed to react at 25°C for 10 min. Following this, 1 mL salicylic acid solution (1.8 mmol/L) was added to the mixture for reaction at room temperature for 30 min. The absorbance was measured at 510 nm using a UV–visible spectrophotometer (Shimadzu, Tokyo, Japan). The percentage clearance effect (%) was expressed as:


$$\mathrm{Hydroxyl radical scavenging activity}=1-\frac{A_{\mathrm{sample}}-A_{\mathrm{control}}}{A_{\mathrm{blank}}}\times 100$$

where *A*_sample_ is the absorbance of the samples in the reaction; *A*_control_ is the absorbance of the salicylic acid; and *A*_blank_ is the absorbance of water (in the absence of the sample).

##### 2.5.1.2 DPPH radical scavenging capacity

A total of 2 mL of aqueous sample solution was added to several stoppered test tubes, followed by 2 mL of DPPH solution at a concentration of 0.04 mg/mL. The solution was then vortexed and mixed. The reaction was performed in the dark for 30 min. Once the reaction was over, the absorbance was measured at 517 nm. The DPPH solution was replaced with 2 mL of methanol in the sample control group, and the sample solution was replaced with 2 mL of distilled water in the blank control group, with reference to the method of Zhao et al. (16). The clearance (%) was calculated as follows:


$$\mathrm{DPPH radical scavenging capacity}\;\left(\%\right)=\frac{A_{1}-A_{0}}{A_{2}-A_{0}}\times 100$$

where *A*_0_, *A*_1_, and *A*_2_ are the absorbances of the blank control, sample, and sample control groups, respectively.

##### 2.5.1.3 ABTS radical scavenging activity

A 100 mL aliquot of a 7 mM ABTS solution and 1.6 mL of 2.45 mM potassium persulfate was mixed and kept overnight in the dark at room temperature for 14 h. The mixture was then diluted with distilled water to obtain the stock solution with an absorbance of 0.7 ± 0.02 at 734 nm. A volume of 0.8 mL of different concentrations (0.5 mg/mL, 1.0 mg/mL, 1.5 mg/mL, 2.0 mg/mL, 2.5 mg/mL) of each sample was mixed with 3.2 mL of the ABTS radical stock solution and incubated at room temperature for 6 min in the dark. The absorbance of the sample was measured at 734 nm and the ABTS radical scavenging activity (%) was calculated as follows:


$$\mathrm{ABTS radical scavenging activity}=\frac{A_{0}-A_{1}}{A_{0}}\times 100$$

where *A*_0_ and *A*_1_ are the absorbances of the blank control and sample groups, respectively.

#### 2.5.2 Cell culture and grouping

HepG2 cells were cultured in MEM medium containing 10% fetal bovine serum, 100 U/mL penicillin, and 100 μg/mL streptomycin in a saturated humidity incubator at 37°C with 5% CO_2_. Three groups were then used for the analysis. The normal group compromised the complete medium. For the oxidative stress model group, a concentration of 300 μmol/L H_2_O_2_ medium solution was added to stimulate the solution for 2 h and the complete medium (same volume as that of the normal group) was then added to incubate for 24 h. For the experimental group, the concentration of 300 μmol/L H_2_O_2_ solution was added to stimulate the solution for 2 h, and the final mass concentrations of 50, 100, 200, and 400 μg/mL of the four proteins were then added, respectively, to culture for 24 h.

#### 2.5.3 Assay for cell viability evaluation

HepG2 cells in the logarithmic growth phase were inoculated into a 96-well cell culture plate at a density of 1 × 10^5^ cells/mL. Cells are processed based on the cell culture and grouping techniques outlined in Section 2.5.2. Cell viability was determined using the CCK-8 method. The cell viability (%) of each group was calculated using the following formula:


$$\mathrm{Cell viability}/\%=\frac{\mathrm{Absorbance of sample group}}{\mathrm{Normal group absorbance}}\times 100$$

#### 2.5.4 Live and dead staining of HepG2 cells

HepG2 cells in the logarithmic growth phase were selected and seeded in 24-well plates with a volume of 0.5 mL per well and an inoculation density of 1.0 × 10^5^ cells/mL. The cells were processed based on the cell culture and grouping techniques outlined in Section 2.5.2. The cells were then washed with PBS 2–3 times and 500 μL of diluted Calcein-AM solution was added to each well. The cells were incubated at 37°C in the dark for 20 min in the incubator. Following this, they were washed three times with PBS and 200 μL of diluted PI solution was added. The cells were then allowed to react in the dark at 37°C for 5 min. Finally, the cells were washed 2–3 times with PBS and observed under a fluorescence microscope.

#### 2.5.5 ROS assay

Logarithmically growing HepG2 cells were selected and seeded in 6-well plates at a density of 1.0 × 10^5^ cells/mL with a volume of 2 mL per well. The cells were processed based on the cell culture and grouping techniques outlined in Section 2.5.2. 2′,7′-Dichlorodihydrofluorescein diacetate (DCFH-DA) fluorescent probes were added to each well at a final concentration of 10 μmol/L. After incubating in the dark at 37°C for 20 min, the probes were removed and the cells were washed three times with pre-cooled PBS, with reference to the method of Hong et al. (24). The cell morphology was evaluated using a fluorescence inverted microscope and the ROS levels were measured using flow cytometer (Beckman Instruments, NanJing, China).

#### 2.5.6 Mitochondrial membrane potential analysis

JC-1 fluorescent dye was used to assess the mitochondrial membrane potential (MMP) according to the method of Hu et al. (25). HepG2 cells in the logarithmic growth phase were selected and seeded in 24-well plates at a density of 1.0 × 10^5^ cells/mL, with a volume of 0.5 mL per well. The cells were processed based on the cell culture and grouping techniques outlined in Section 2.5.2. Changes in the cell fluorescence color in the different groups were observed using the mitochondrial membrane potential detection kit.

#### 2.5.7 Annexin V-FITC/PI apoptosis assay

HepG2 cells in the logarithmic growth phase were selected and inoculated into 6-well plates with a volume of 2 mL per well and an inoculation density of 1.0 × 10^5^ cells/mL. The cells were cultured in an incubator at 37°C and 5% CO_2_. After 2 h of H_2_O_2_ modeling, the four proteins were added and cultured for 24 h. The apoptosis of HepG2 cells in different groups was analyzed and detected by flow cytometry using an apoptosis kit.

#### 2.5.8 Malondialdehyde, lactate dehydrogenase, superoxide dismutase, catalase, and glutathione peroxidase measurements

The activities of superoxide dismutase (SOD), catalase (CAT), and glutathione peroxidase (GSH-Px), dehydrogenase (LDH) and malondialdehyde (MDA) were measured with kits (Sino Best Biological Technology Co., Ltd., Shanghai, China). In brief, following the treatment, HepG2 cells were collected and homogenized with lysis buffer and centrifuged for 20 min at 12,000 rpm and 4°C. The malondialdehyde (MDA), lactate dehydrogenase (LDH), SOD, CAT, and GSH-Px levels of the supernatant were detected according to the manufacturer’s instructions.

#### 2.5.9 Statistical analysis

All experiments were conducted at least three times. Data are reported as the mean ± standard deviation. Statistical analyses were conducted using SPSS 21.0 (IBM Corp.) and Origin 9.0 (Origin Lab) for Windows. Values of *p* < 0.05 were considered statistically significant.

## 3 Results and discussion

### 3.1 Screening to find the best *Ginseng-Schisandra* ratio

The optimal ratio of *Ginseng* and *Schisandra* was determined through electrophoresis and free radical scavenging experiments. Supplementary Figure S1A shows that SDS–PAGE analysis revealed darker bands at the 6:1 ratio. In addition, the DPPH and ABTS free radical scavenging ability of *Ginseng*-*Schisandra* protein was the highest at the 6:1 ratio, at 89.39 and 45.11%, respectively (Supplementary Figure S1B). Thus, the protein at the 6:1 *Ginseng*-*Schisandra* ratio was selected as the COP of *Ginseng*-*Schisandra* for analysis. The SMP was used as the control to analyze and compare the effects of the two mixing methods on the physical and chemical properties and biological activity of the protein.

### 3.2 Physicochemical properties

#### 3.2.1 SDS–PAGE

**Figure 1:**
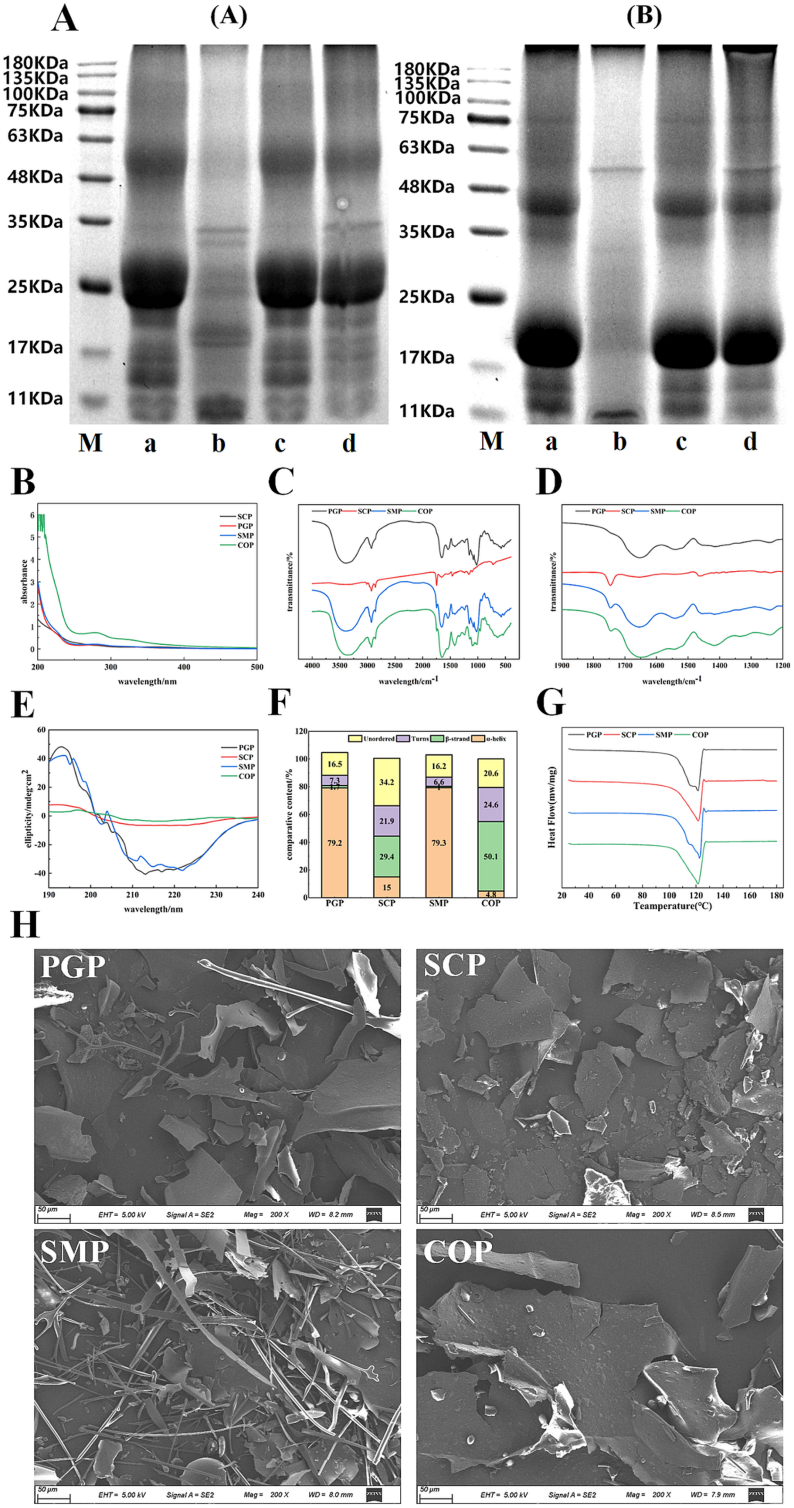
Physicochemical properties of four proteins. **(A)** SDS-PAGE, **(B)** UV **(C)** FTIR **(D)** The 1,900–1,200 cm^−1^ magnification map of FTIR **(E)** CD **(F)** Secondary structure content **(G)** DSC **(H)** SEM. M is Maker; a is PGP; b is SCP; c is SMP; d is COP. In **(A)** is reductive electrophoresis and **(B)** is nonreductive electrophoresis. SDS-PAGE, Sodium dodecyl sulfate–polyacrylamide gel electrophoresis; UV, Ultraviolet spectra; FTIR, Fourier transform infrared spectroscopy; CD, Circular dichroism spectra; DSC, Differential scanning calorimetry; SEM, Scanning electron microscopy; PGP, Ginseng protein; SCP, Schisandra protein; SMP, Single mixed proteins; COP, Co-extracted protein.

Figure 1A reveals that during non-reducing electrophoresis, numerous soluble aggregates in COP were obstructed at the top of the separated gel. This may be due to the co-extraction of some *Ginseng* and *Schisandra* proteins into the aggregates, resulting in the formation of disulfide bonds between PGP and SCP, which altered their subunit composition. Compared to reducing electrophoresis, the bands of COP and SMP in non-reducing electrophoresis shifted downwards, with bands appearing at 20 KDa and 35–48 KDa. Additionally, in non-reducing electrophoresis, *Schisandra* protein and co-extracted protein appeared at approximately 60 KDa, while in reducing electrophoresis, *Schisandra* protein and co-extracted protein appeared at approximately 34 KDa. In contrast, single mixed proteins did not appear in these molecular weight ranges. The lower Schisandra protein content in SMP indicates that the two proteins can be fully mixed using co-extraction technology. Compared with the molecular weight distribution of SMP, COP exhibited minimal changes. This agrees with the findings by Tian in soybean-wheat co-precipitated proteins (26). We also observed the subunit band of the PGP-SCP combination in the subunit band of COP, demonstrating that the intact primary structures of proteins were retained. This is consistent with the results of He et al. (11) and Cui et al. (27) and indicates that co-extraction technology can fully mix the two proteins, revealing the advantages of the co-extracted proteins.

#### 3.2.2 Ultraviolet spectra

The maximum absorption peaks of the four proteins were observed close to 280 nm (Figure 1B). This is attributed to the absorption of chromophore amino acid residues such as tyrosine and tryptophan within the proteins, which is similar to the unique absorption peak of Grass pea protein at 270–275 nm observed by Mahmood et al. (28). Proteins contain aromatic amino acids (e.g., tyrosine, tryptophan, and phenylalanine), which can absorb UV light at 280 nm (29). COP exhibited the highest UV absorption intensity near 280 nm, indicating that the aromatic amino group exhibits the highest reactivity, including Try and Trip. This shows that COP is rich in amino acid content, which is likely transferred from the internal hydrophobic region to the polar solvent environment during co-extraction, thus increasing the UV absorption intensity. The folding and aggregation of the SMP molecules may reduce the exposed chromophores and, consequently, the UV absorption intensity. SCP exhibited the weakest absorption intensity, possibly due to its low protein content. During the co-extraction process, the disulfide bond cleavage may have resulted in the formation of small molecular peptides, resulting in an absorption peak in COP at 200–220 nm.

#### 3.2.3 Fourier transform infrared spectroscopy

All four proteins exhibit three spectral bands at approximately 1,560, 1,650, and 3,400 cm^−1^ (Figures 1C,D), which are associated with N-H, C-O, and OH stretching, respectively. A band near 3,250 cm^−1^ is also observed in the four proteins, which may be attributed to the N-H stretching of aliphatic primary amines. Furthermore, the four proteins exhibit a broad and intense band at 3,600–3,100 cm^−1^, which is typically associated with the N-H stretching vibration of protein molecules and their intermolecular hydrogen bonding, as well as the N-H bonding of amides. The peaks of SCP are smaller and smoother compared to those of the other three proteins, possibly due to its lower protein content and fewer hydrogen bonds. All four proteins exhibit bands at 3,100–3,000 cm^−1^, indicating the presence of unsaturated hydrocarbon (=C-H) stretching vibrations in the aromatic ring, likely due to the presence of aromatic amino acid residues. The peak positions of SMP and COP at 2,930 cm^−1^ remained almost unchanged, indicating that the extraction method had a minimal effect on the stretching vibration of the C-H bond of the proteins. The differences in absorption peaks within the infrared absorption spectra of the four proteins reveal differences in their internal structures, which are important factors leading to differences in their physicochemical and functional properties.

#### 3.2.4 Circular dichroism spectroscopy

Figure 1E depicts the far-ultraviolet CD spectra of the four proteins. All four proteins displayed positive bands near 195 nm and wide negative bands with a minimum ranging from 210 to 225 nm. PGP and SMP exhibited more negative band ellipticities, indicating a higher content of the *α*-helical structure. The CD spectra of PGP and SMP were similar, suggesting that COP was a mixture of just these two proteins. In contrast, the peak difference between COP and SMP was larger, suggesting that COP underwent protein changes during the extraction process to obtain a co-extracted complex protein.

Figure 1F reveals the considerable differences in the secondary structure composition of COP and SMP, indicating that the extraction method has a strong impact on the structural changes of the complex proteins. SCP exhibited more random curls with COP compared to PGP and SMP (at 34.2 and 20.6%, respectively). The co-extraction of the two proteins may cause the other ordered protein structures to transform into irregularly curled structures. The *β*-folding content was the highest (50.1%), while the *α*-helix content was the lowest (4.8%). The percentage of the β-fold content was found to be the highest (91.8%) in COP, while α-helix content was the highest (79.3%) in SMP. This may be due to the partial de-folding of the α-helical regions of the complex proteins extracted by the co-extraction method, reducing the α-helix content and increasing the β-fold content. These findings suggest that the protein co-extraction method can improve the functional properties of the protein by altering its secondary structure.

#### 3.2.5 Differential scanning calorimetry

The thermal properties of the four proteins were determined by DSC (Figure 1G). PGP had a thermal denaturation temperature of 128°C, while the remaining three proteins had a temperature of 122°C. The thermal properties of the four proteins were similar. The absorption peak of COP had a lower peak value of −263.373 mw/mg, which may be due to the smaller surface charge of the aggregates formed by COP. Consequently, the spatial resistance of COP decreased, resulting in protein aggregation. The thermal stability of proteins improves with higher denaturation temperatures. The thermal stability of COP did not differ considerably from the other three proteins. This reveals the potential of COP in replacing single proteins in food products, as the thermal stability of co-extracted proteins is an indicator of their thermal changes in various food processing systems.

#### 3.2.6 Scanning electron microscopy

Figure 1H shows the structures of the four proteins observed under SEM. PGP and SMP exhibit a striped structure, indicating the presence of fibrous structures. SMP contains a considerable amount of fibrous structure, likely due to the network of shorter fibers in PGP, which results in the formation of a strong fibrous structure when mixed with SCP. The striped protein molecules of PGP are integrated into the fibrous structure of SCP, resulting in a wider gap between the causal groups. This may be attributed to the incomplete fusion of PGP and SCP during the mono-mixing process, which was not observed in COP. Furthermore, the strip structure of PGP and the block structure of SCP were no longer present in COP. Instead, a new lamellar structure possibly the aggregation unit produced by co-extraction emerged. This lamellar structure exhibited multi-layer bending in space, facilitating folding and texture formation to enhance adsorption. This is also why COP exhibited superior water and oil retention properties compared to the other three proteins.

#### 3.2.7 Protein solubility analysis

**Figure 2:**
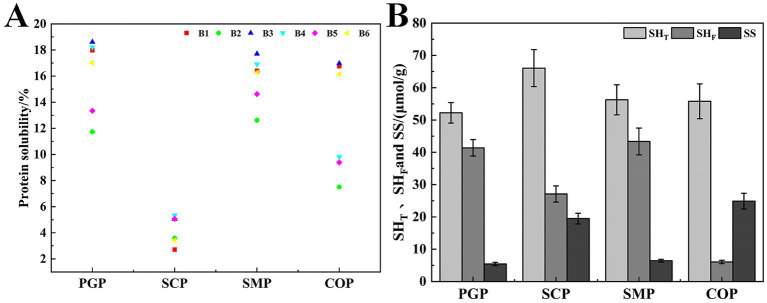
Analysis of interaction forces between complex proteins. **(A)** Protein–protein interaction. **(B)** Free sulfhydryl groups (SHF), total sulfhydryl groups (SHT), and disulfide bond (SS).

The proteins were solubilized using different methods based on the six solutions described in Section 2.3.7. SDS was used to disrupt hydrogen bonding and hydrophobic bonding interactions, urea was used to disrupt hydrogen bonding, and *β*-mercaptoethanol was used to cleave disulfide bonding and sulfhydryl groups (30). Compared to the other three proteins, COP had lower hydrophobicity (Figure 2A) as the interaction between PGP and SCP formed insoluble aggregates during co-extraction. This also resulted in higher disulfide bonding in COP, indicating that both proteins denatured during co-extraction and shifted free sulfhydryl groups to disulfide bonding. These findings suggest that hydrogen and disulfide bonding were the main forces in the co-extraction process. A high similarity was observed between SMP and PGP, indicating that there is no chemical reaction between these two proteins in the single-mixing process.

#### 3.2.8 Free sulfhydryl groups, total sulfhydryl groups, and disulfide bond contents

Figure 2B shows that the disulfide bond content of COP obtained after co-extraction was 24.547%, which is higher than that of SCP (16.611%), SMP (3.916%), and PGP (1.154%). COP had the lowest free sulfhydryl group content at 6.022%. This may be due to the unfolding of the folded structure after co-extraction, the increase in the reactivity of the exposed free sulfhydryl group, and the oxidation of the sulfhydryl group to form disulfide bonds. These factors reduced the content of the free sulfhydryl group. Furthermore, the high content of disulfide bonds in COP may be attributed to the accelerated protein unfolding rate during co-extraction. This exposes more free sulfhydryl groups and enhances intermolecular interactions, leading to the conversion of free sulfhydryl groups into disulfide bonds. These findings indicate that the structure of COP is highly aggregated. We determined that the content of the sulfhydryl and disulfide bonds of the protein changed after co-extraction, which is consistent with the results of Tian et al. (31).

#### 3.2.9 Amino acids composition

All four proteins were determined to have a complete range of 16 amino acids (Table 1). The amino acid content of SMP was significantly lower than that of COP. This suggests that the single-mixing method can only mix two proteins individually without any chemical reaction occurring. Compared with SMP, the amino acid content of COP increased from 14.04 to 26.98% by the co-extraction method, effectively increasing the amino acid content. This is similar to the higher nutritional value of milk co-precipitates reported by Alu’datt et al. (15). This may be a characteristic reflection of the complementary effect of the Chinese herbal medicine pair pairing. In addition, the glutamic acid, arginine, and aspartic acid contents in COP were significantly higher than those in SMP, suggesting better biological activity.

**Table 1:** Amino acid composition and content of the four proteins.

Name	SCP (g/100 g)	COP (g/100 g)	SMP (g/100 g)	PGP (g/100 g)
MetSON	8.160 ± 0.190	49.587 ± 0.810	22.070 ± 0.320	43.071 ± 0.770
Threonine (Thr)	3.017 ± 0.080	17.069 ± 0.200	11.048 ± 0.170	16.801 ± 0.310
Serine (Ser)	5.649 ± 0.120	16.157 ± 0.190	10.743 ± 0.160	14.087 ± 0.260
Glutamic acid (Glu)	13.987 ± 0.510	43.223 ± 0.760	19.409 ± 0.290	32.320 ± 0.370
Glycine (Gly)	28.046 ± 0.430	13.288 ± 0.150	7.414 ± 0.120	10.004 ± 0.150
Alanine (Ala)	19.756 ± 0.220	20.28 ± 0.290	10.443 ± 0.190	20.785 ± 0.310
Valine (Val)	2.217 ± 0.070	9.972 ± 0.110	3.599 ± 0.930	8.112 ± 0.180
Methionine (Met)	0.332 ± 0.008	1.533 ± 0.030	0.743 ± 0.004	0.977 ± 0.010
Isoleucine (Ile)	1.476 ± 0.020	10.339 ± 0.180	4.594 ± 0.110	8.727 ± 0.190
Leucine (Leu)	3.515 ± 0.060	19.798 ± 0.280	8.663 ± 0.130	15.799 ± 0.290
Tyrosine (Tyr)	1.622 ± 0.030	8.099 ± 0.160	3.088 ± 0.070	5.693 ± 0.150
Phenylalanine (Phe)	2.235 ± 0.030	13.318 ± 0.230	8.009 ± 0.150	9.867 ± 0.220
Histidine (His)	1.291 ± 0.020	6.635 ± 0.090	4.587 ± 0.100	4.658 ± 0.110
Lysine (Lys)	3.798 ± 0.090	12.421 ± 0.230	8.775 ± 0.110	11.248 ± 0.190
Arginine (Arg)	6.587 ± 0.110	16.955 ± 0.280	7.589 ± 0.130	8.102 ± 0.190
Proline (Pro)	14.975 ± 0.280	11.149 ± 0.210	9.666 ± 0.190	12.924 ± 0.240
total (ug/mg)	116.664 ± 2.268	269.823 ± 4.200	140.44 ± 3.174	223.175 ± 3.940
Content %	11.6664 ± 0.020	26.9823 ± 0.040	14.044 ± 0.030	22.3175 ± 0.040

### 3.3 Functional properties

#### 3.3.1 Solubility

**Figure 3:**
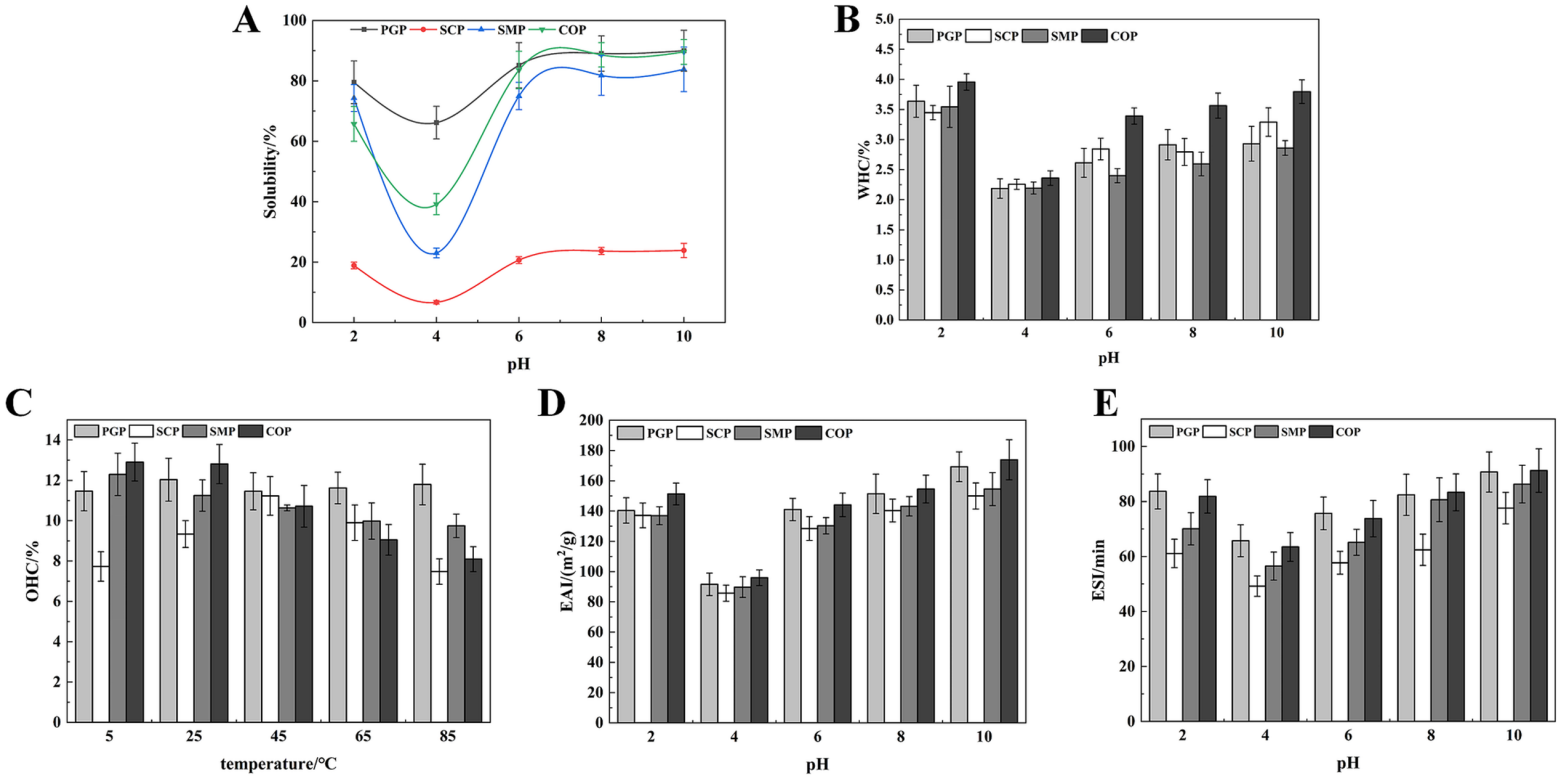
Functional properties. **(A)** Solubility **(B)** WHC **(C)** OAC **(D)** EAI **(E)** ESI. WHC, Water holding capacity; OAC, Oil adsorption capacity; EAI, Emulsification efficiency; ESI, Emulsion stability.

Solubility is a key characteristic in food systems, as it determines the effective utilization of proteins in food applications (18). At the pH of 4, which is close to the isoelectric point of the four proteins, there was minimal intermolecular charge repulsion, resulting in the lowest protein solubility (Figure 3A). SCP exhibited the lowest solubility at 6.70%. When the proteins were placed in either acidic or alkaline environments that deviated from the isoelectric point, their solubility tended to increase, with higher solubility in alkaline environments compared to acidic environments. This can be attributed to the increase in the net negative and positive charges as the pH deviates from the isoelectric point, which promotes intermolecular repulsion, enhances the dispersive ability, and ultimately increases protein solubility (32). The solubility of the four proteins was highest at pH 10, with PGP exhibiting the highest solubility at 89.97%, followed by COP at 89.57%, SMP at 83.81%, and SCP at 23.87%. The solubility of COP was lower than that of PGP, which may be due to the formation of insoluble aggregates during the co-extraction process, resulting in a decrease in solubility.

#### 3.3.2 Water holding capacity

The WHC of all four proteins was lowest at pH 4 and increased with pH (Figure 3B). This is likely due to the proteins existing in ionic form when the pH is far from the isoelectric point, which enhances the proteolytic properties, viscosity, and WHC. The WHC of COP increases with the pH. This is similar to the improved WHC of soybean-wheat co-precipitated proteins found by Tian et al. (26). COP had the strongest WHC (3.80%). This indicates that COP has the most hydrogen bonding interactions and hydrophilic groups exposed under alkaline conditions.

#### 3.3.3 Oil adsorption capacity

COP exhibited strong OAC at low and room temperatures, with 12.90% at 5°C and 12.80% at 25°C (Figure 3C). This is likely due to the exposure of more hydrophobic groups during the co-extraction process, which can interact with oil molecules and improve oil-holding properties. The OAC of SCP exhibited an increasing and subsequently decreasing trend with rising temperature, with the highest OAC of 11.23% at 45°C. This decrease in OAC may be attributed to the denaturation of SCP at higher temperatures, which increased the hydrophilic groups. The superior OAC of COP compared to SMP indicates that COP’s high oil adsorption capacity can serve as an excellent additive for high-fat food products (e.g., meat and sausage) to enhance their taste and flavor.

#### 3.3.4 Foaming properties and foaming stability

The EAI and ESI of all proteins initially decreased and then increased at all pH values (Figures 3D,E). The lowest values of EAI and ESI were observed at pH 4 for all proteins. This is because pH 4 was the isoelectric point, and protein aggregation occurred close to this point. As the pH increases, the protein will be degraded, and the exposed hydrophobic amino acid residues point to the oil phase, while the hydrophilic groups point to the water phase, thereby enhancing the interaction with the oil phase (33). Therefore, the emulsifying properties increased with the pH value. In addition, COP exhibited high EAI (173.92m^2^/g) and ESI (91.23min) values. Thus, COP, characterized by superior emulsifying capabilities and emulsion stability, holds considerable potential in enhancing food processing attributes. This is consistent with Al-Saadi and Deeth (34), who reported the excellent emulsifying activity and stability of protein co-precipitate from sheep milk.

### 3.4 Free radical scavenging activity

**Figure 4:**
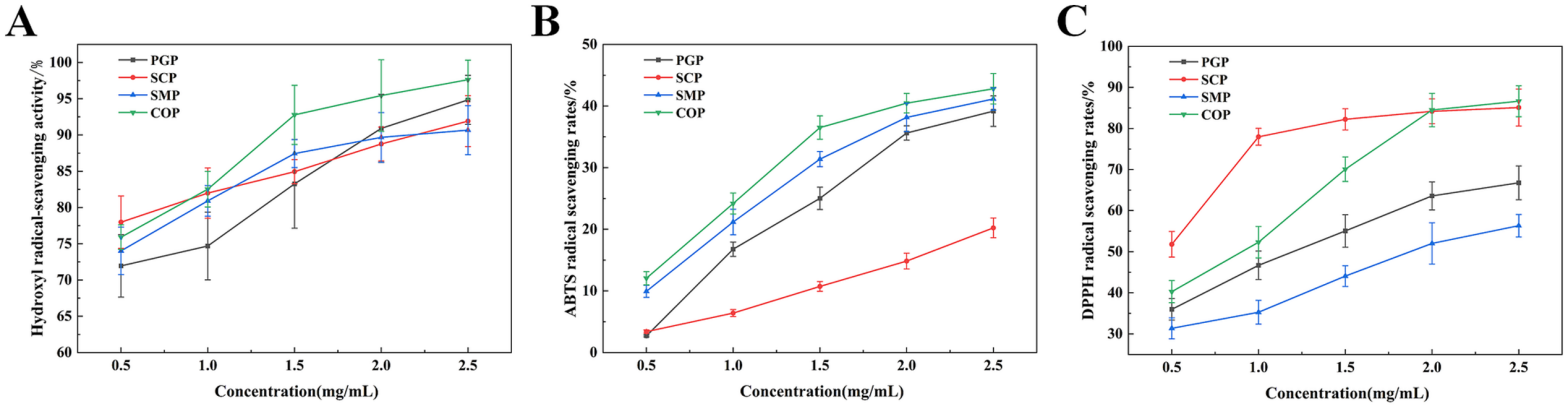
Free radical scavenging activity. **(A)** Hydroxyl radical-scavenging activity. **(B)** DPPH radical scavenging capacity. **(C)** ABTS radical scavenging activity. DPPH, 1,1-Diphenyl-2-picrylhydrazyl radical; ABTS, 2,2-biazobis (3-ethylbenzothiazoline-6-sulfonic acid) diamine salt.

The hydroxyl radical is the most active free radical among the three reactive oxygen species. It can easily enter the cell membrane, react with biological macromolecules, and cause tissue damage (35). Figure 4A demonstrates that all four proteins exhibit hydroxyl radical scavenging ability, which is proportional to concentration. COP has the highest scavenging ability (98.89%) at a concentration of 2.5 mg/mL, while SMP has the lowest scavenging ability (90.88%). This suggests that the co-extraction can alter the properties of the two proteins.

The DPPH-scavenging ability of the two proteins significantly increased after extraction using the co-extraction method (Figure 4B) (36). The DPPH radical scavenging activities of the four protein samples also increased with the sample concentration in the range of 0.5–2.5 mg/mL. The enhancement was more rapid within the concentration range of 0.5–1.5 mg/mL, while it tended to level off between concentrations of 1.5 and 2.5 mg/mL. The optimal scavenging effect of COP was achieved at a concentration of 2.5 mg/mL, resulting in an 85.95% reduction in DPPH radicals. This suggests that COP is a promising scavenger of free radicals.

Figure 4C reveals the ABTS scavenging activity of the four proteins was positively correlated with the sample concentration in the range of 0.1–2.5 mg/mL and increased with the sample concentration. COP had the highest ABTS free radical scavenging ability (42.69 mg/mL) at 2.5 mg/mL. Therefore, COP has potential as an antioxidant due to its strong free radical scavenging ability.

### 3.5 Evaluation of antioxidant activity in the H_2_O_2_-induced HepG2 cell model

#### 3.5.1 Effect on the growth of HepG2

Four protein concentrations (50, 100, 200, and 400 μg/mL) and an H_2_O_2_ modeling condition of 300 μmol/L were selected for further analysis for 2 h (Supplementary Figure S2). The repairing effects of the four proteins on H_2_O_2_-induced cellular damage were compared. COP was found to exhibit improved repairing effects on oxidative stress. This indicates its potential to repair H_2_O_2_-induced oxidative stress damage in HepG2 cells.

#### 3.5.2 Intracellular oxidative stress levels

**Figure 5:**
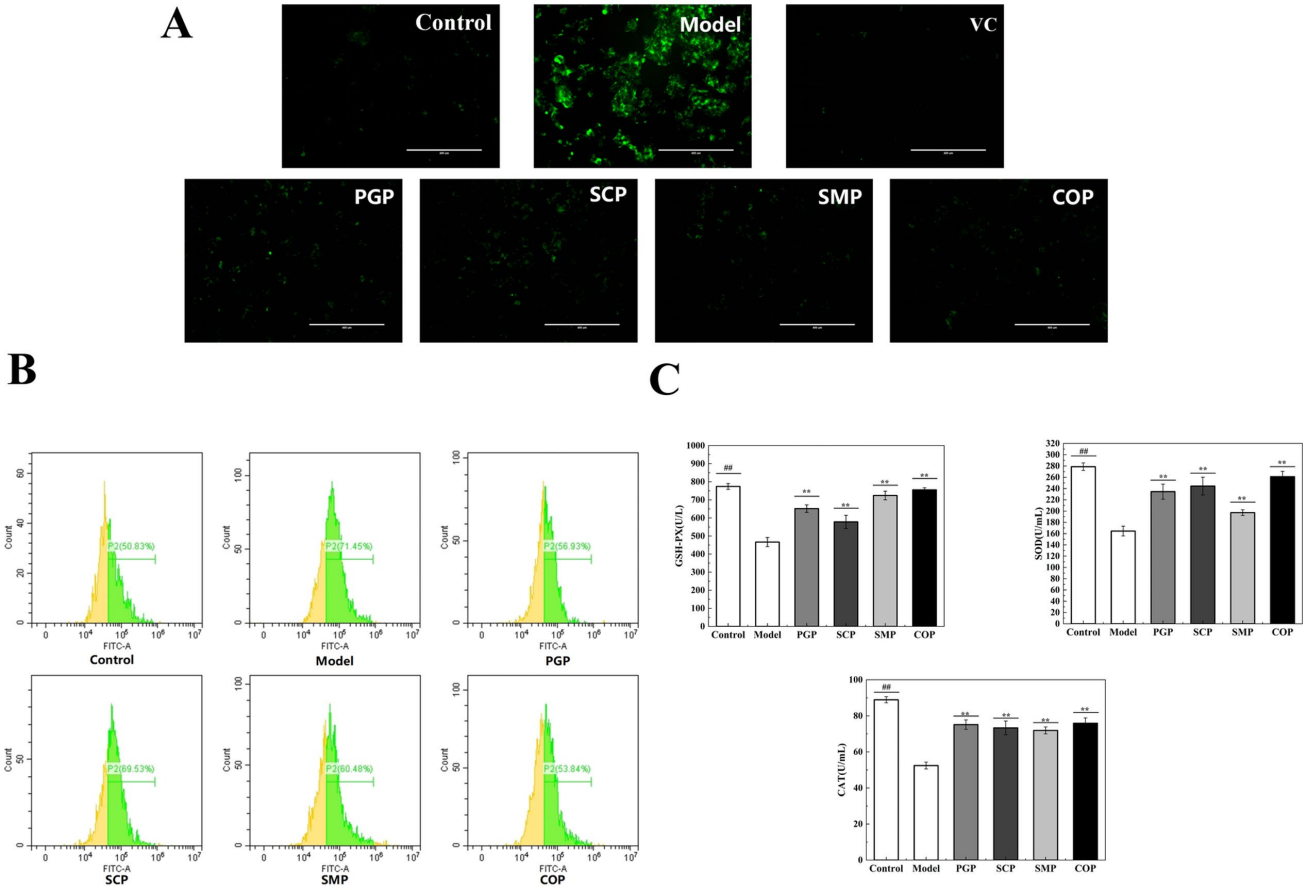
Evaluation of anti-oxidative stress. **(A)** Staining of intracellular ROS. **(B)** Evaluation of ROS by flow cytometry. **(C)** Effect on Effect of anti-oxide enzyme activity (*p < 0.05, **p < 0.01, and ***p < 0.001). GSH-PX, Glutathione peroxidase; SOD, Superoxide; CAT, Catalase.

Green fluorescence was significantly higher in the model group compared to the blank group (Figure 5A). SCP exhibited higher levels of ROS, while COP displayed the lowest green fluorescence, indicating its superior antioxidant activity. Figure 5B shows that ROS levels significantly increased in the model group compared to the blank group. Four proteins were found to effectively alleviate cellular ROS levels. Among them, COP had the strongest effect, which was similar to the blank group. This is consistent with the staining results.

To study the effects of the four proteins on oxidative stress levels, we determined the effect of antioxidant enzyme activities on the protective mechanism. SOD catalyzes the decomposition of superoxide anion into H_2_O_2_ and O_2_, thereby scavenging free radicals. CAT catalyzes the decomposition of H_2_O_2_ into H_2_O by scavenging excess free radicals produced in the body, thus maintaining the body’s redox balance (37). GSH-Px can catalyze GSH into oxidized glutathione (GSSG), reduce peroxides into hydroxyl compounds, and promote the decomposition of H_2_O_2_ with CAT to protect the structure and function of the cell membrane (38). Figure 5C depicts the activities of SOD, CAT, and GSH-Px. After 2 h of exposure to H_2_O_2_, the activities of SOD, CAT, and GSH-Px were significantly reduced in the HepG2 cells compared to the normal group. Moreover, the levels of SOD, CAT, and GSH-Px were significantly higher in the group treated with the four proteins compared to the model group.

#### 3.5.3 Effects on cell survival

**Figure 6:**
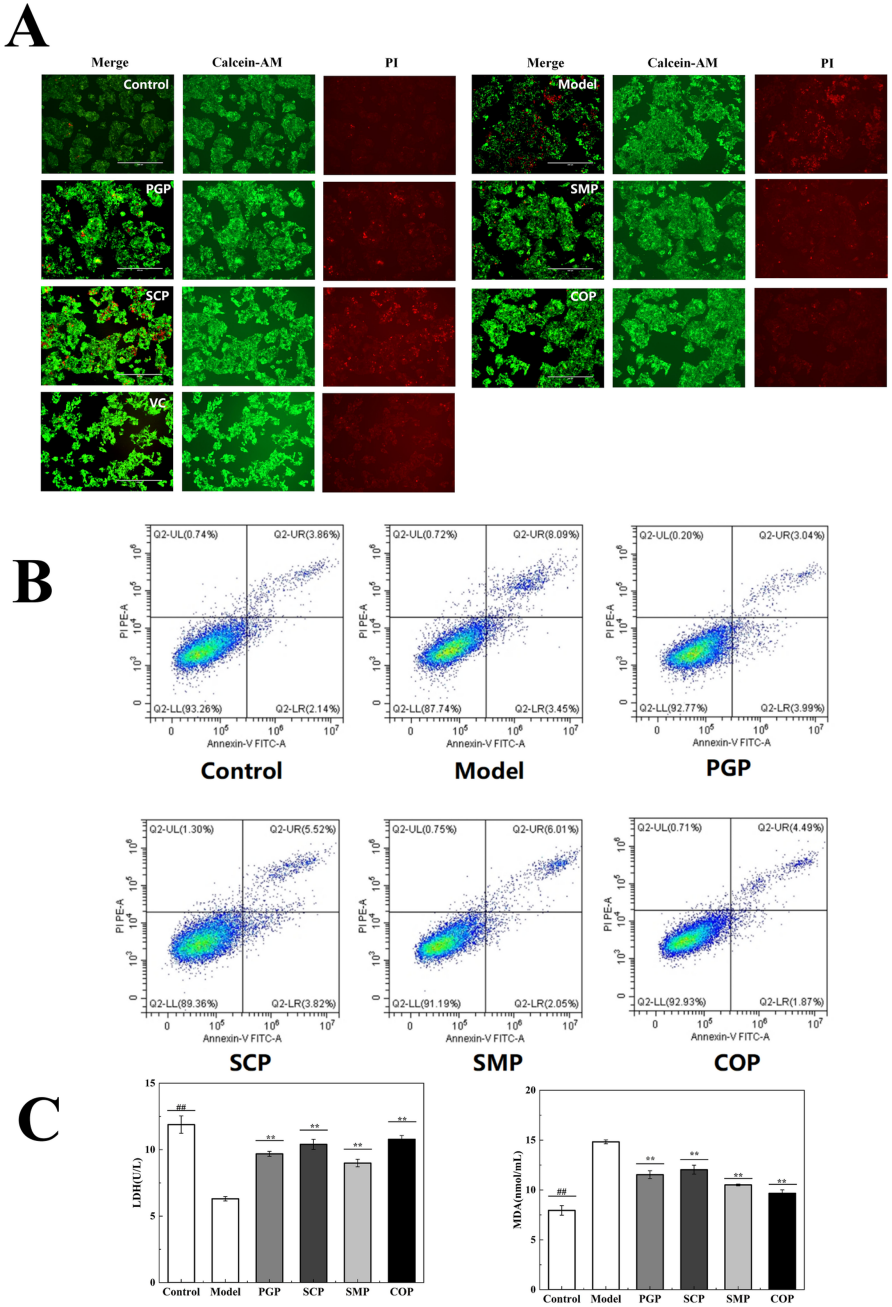
Evaluation of cell injury. **(A)** Calcein-AM/PI dual-color fluorescent apoptosis staining. **(B)** Apoptosis was assessed using An-nexin V-FITC/PI staining. **(C)** Effect on MDA and LDH activities (*p < 0.05, **p < 0.01, and ***p < 0.001). MDA, malondialdehyde; LDH, lactate dehydro-genase.

Figure 6A reveals that cells in the model group presented wrinkles, a lower cell density, weaker green fluorescence, and relatively obvious red nuclei compared to the blank group. These results suggest that the H_2_O_2_-induced oxidative stress in HepG2 cells promotes apoptosis. The addition of the four proteins resulted in significantly weaker red fluorescence and enhanced green fluorescence. Among them, the red fluorescence of COP was the weakest, similar to that of the blank group. This suggests that COP has a considerable reparative effect on oxidative stress and can restore the H_2_O_2_-induced morphological changes in HepG2 cells.

Figure 6B presents the survival of the cells in each group. The model group exhibited a significant increase in apoptotic cells compared to the blank group due to the presence of the H_2_O_2_ treatment. The number of apoptotic cells decreased after all four protein interventions, with the lowest number observed after COP intervention, among them most cells survived similarly to the normal group.

We measured the levels of MDA and LDH in the HepG2 cells to investigate the protective mechanisms of the four proteins against H_2_O_2_-induced cellular damage (Figure 6C). The results demonstrate that MDA and LDH activities were significantly lower in the model group compared to the blank group. Compared with the other three proteins, COP treatment resulted in the lowest MDA and highest LDH levels (9.67 μmol/mL and 10.78 μmol/mL, respectively), indicating its potential to scavenge ROS.

#### 3.5.4 Effects on cellular mitochondrial function

**Figure 7:**
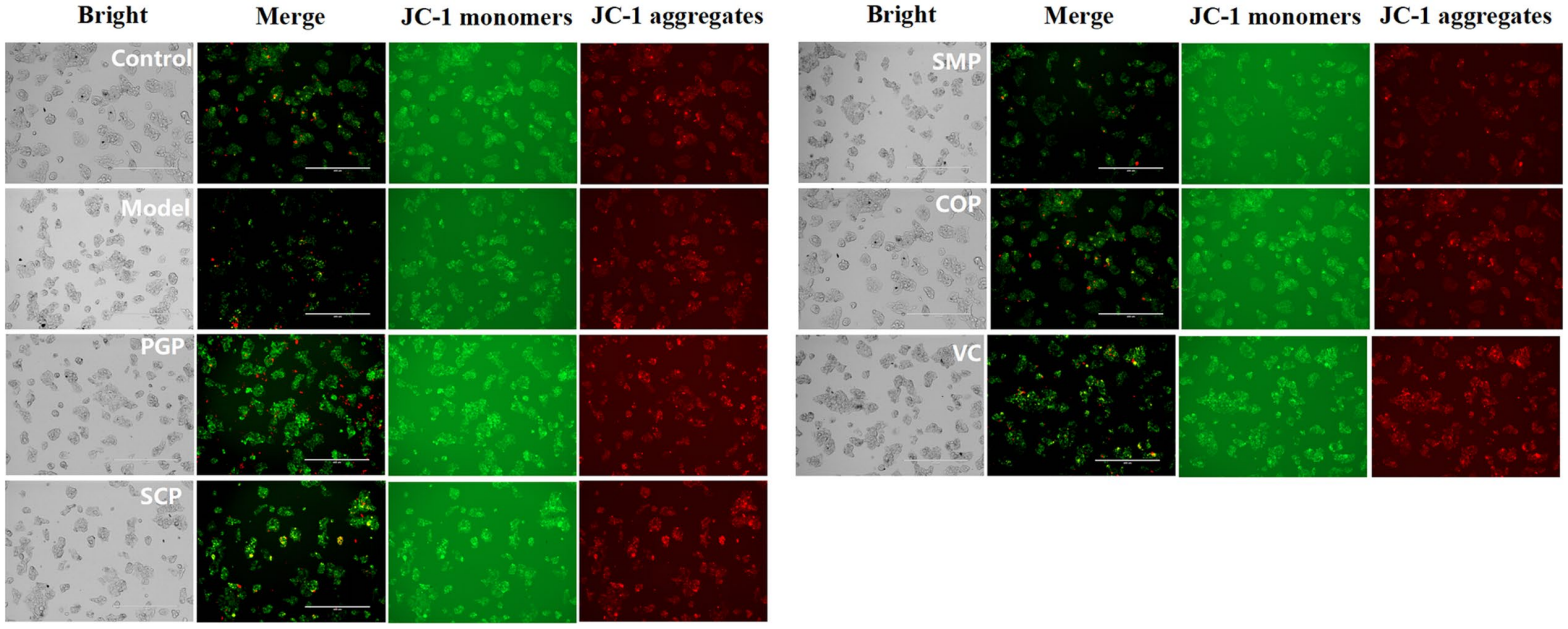
Evaluation of mitochondrial function. The mitochondrial membrane potential was assessed using JC-1 staining at a magnification of 400 μm.

H_2_O_2_ treatment can induce an increase in mitochondrial membrane permeability, which can be detected with JC-1-stained MMP reduction (39). Figure 7 reveals a significant reduction in the mitochondrial membrane potential in the H_2_O_2_-induced group compared to the control group, indicating a decrease in the potential gradient across the mitochondrial membrane and the subsequent impairment of membrane integrity. The four protein groups were able to increase mitochondrial membrane potential and decrease oxidative damage in HepG2 cells. Among these proteins, COP exhibited the highest efficacy in increasing mitochondrial membrane potential, suggesting superior activity compared to the other proteins. Therefore, COP is considered to have better activity.

## 4 Conclusion

There has been growing interest in functional foods in recent years, leading to extensive research in this field. In particular, increasing attention has been directed toward the exploration of novel protein sources, with double proteins emerging as a promising new research avenue. In this study, COP was synthesized using a co-extraction technique, and a comparative analysis of its physicochemical, functional properties, and antioxidant capacities were compared to single proteins and SMP. The findings revealed that the co-extraction process induced structural modifications in the protein, resulting in a reduction in the disulfide bond content of COP, while enhancing its amino acid content by 12.94% compared to SMP. This resulted in the formation of a unique composite protein structure characterized by a denser core and a smooth lamellar morphology, distinct from the fibrous structure observed in SMP under SEM.

We identified the presence of synergistic effects in the co-extracted proteins, with COP exhibiting superior functional properties, including enhanced solubility and emulsification, compared to SMP. Notably, COP exhibited higher free radical scavenging activity, which may be attributed to its increased antioxidant amino acid content. In addition, COP showed restorative effects in an oxidative stress model, with altered levels of MDA, LDH, SOD, CAT, and GSH-PX, confirming its applicability in food processing applications.

Overall, the comparative study confirmed the synergistic effect of COP, which exhibits promising physicochemical properties, high nutritional quality, and excellent biological properties, thus indicating its potential as a high-protein nutritional supplement. Further studies will focus on the synergistic effects exhibited by the co-extracted proteins in terms of biological activities. The study provides a reference for the application of COP in functional foods, aids in improving protein processing technology and adjusting its structural and nutritional characteristics, and supports the application of dual-protein systems and co-extraction technology in the food field.

## Data Availability

The original contributions presented in the study are included in the article/Supplementary material, further inquiries can be directed to the corresponding authors.
