# Supplementary material for: Synergistic effect in the co-extraction of Ginseng and Schisandra protein
Source: Front Nutr. 2024 Oct 31;11:1482125. doi: 10.3389/fnut.2024.1482125 (PMC11562853; doi:10.3389/fnut.2024.1482125)
Supplement: Supplementary file 1 [file Image_1.pdf]

Supplementary Material

Supporting figures

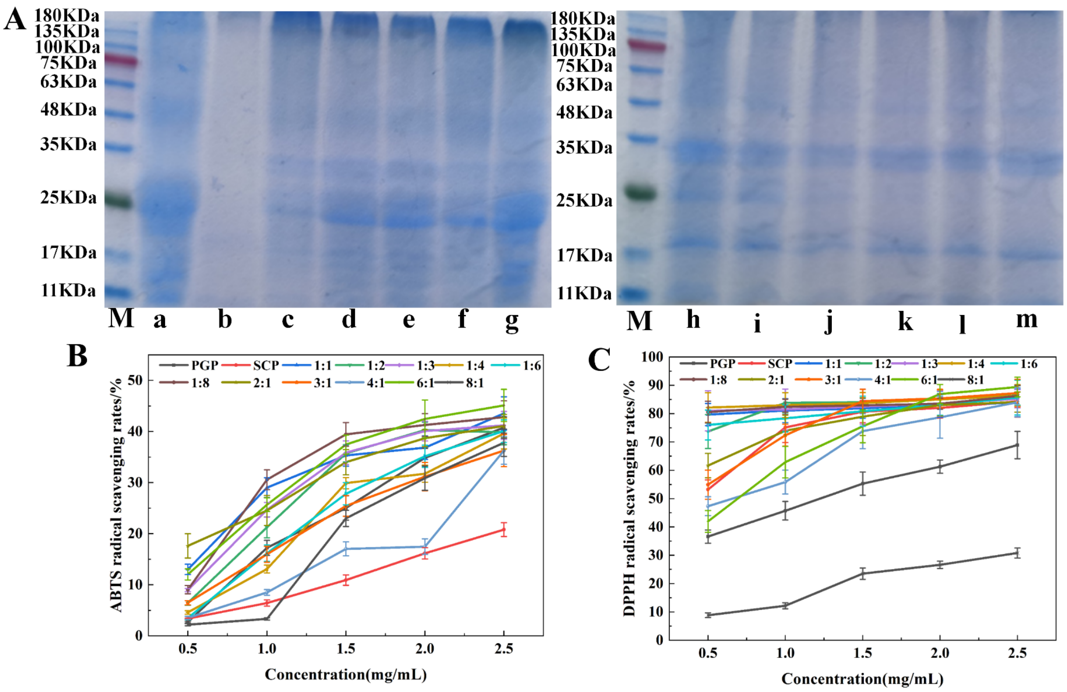

Fig. S1 Screening to find the best Ginseng-Schisandra ratio.(A) SDS-PAGE,(B) DPPH radical scavenging rates,(C) ABTS radical scavenging rates, Note: M is Maker; a is PGP; b is SCP; c is 2:1; d is 3:1; e is 4:1; f is 6:1; g is 8:1; h is 1:1; i is 1:2; j is 1:3; k is 1:4; l is 1:6; m is 1:8.

* Sodium dodecyl sulfate–polyacrylamide gel electrophoresis (SDS-PAGE) * 2,2'-azino-bis-(3-ethylbenzothiazoline-6-sulfonic acid) (ABTS)
* 1,1-diphenyl-2-picrylhydrazyl (DPPH)

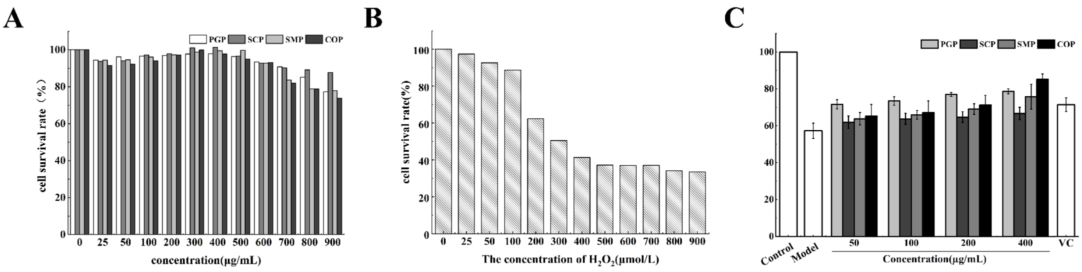

Fig. S2 Antioxidant Activity in the H_2O_2-Induced HepG2 Cell Model (A) Effect on the growth of HepG2 (B) Establishment of cell oxidative damage model (C) Repairing effects on H_2O_2-induced cell injury
