# Supplementary figures and images for: Synergistic effect in the co-extraction of Ginseng and Schisandra protein
Source: Front Nutr. 2024 Oct 31;11:1482125. doi: 10.3389/fnut.2024.1482125 (PMC11562853; doi:10.3389/fnut.2024.1482125)

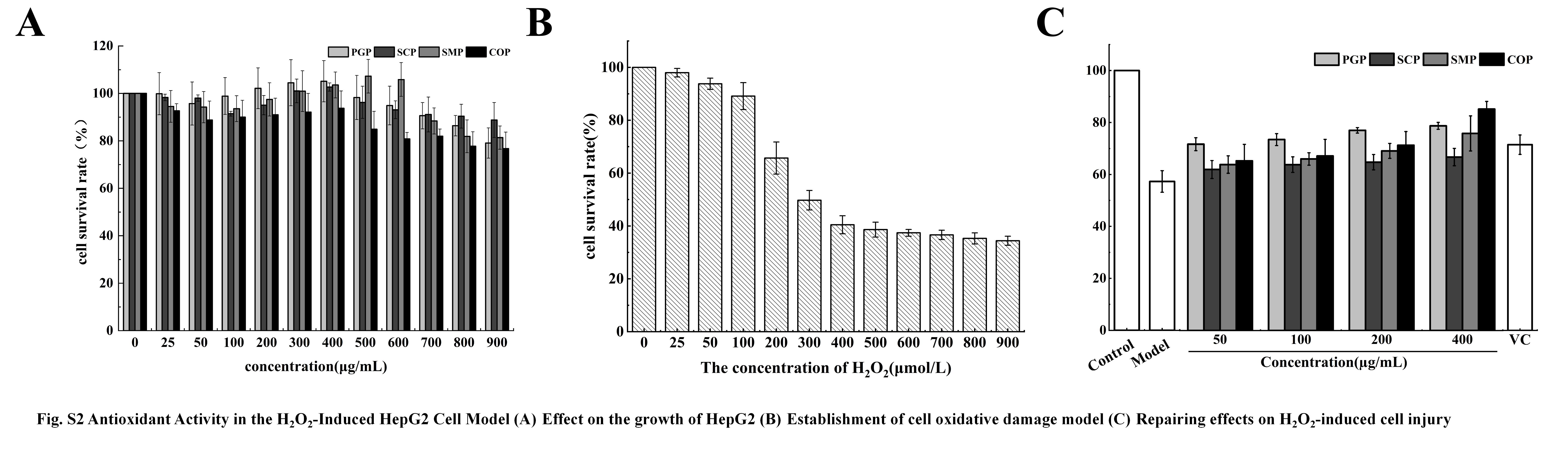

Supplement: Supplementary file 2 [file Image_1.jpg]
